# After Online Innovators Receive Performance-Contingent Material Rewards: A Study Based on an Open Innovation Platform

**DOI:** 10.3390/bs14080723

**Published:** 2024-08-19

**Authors:** Ying Chu, Guijie Qi, Kaiping Wang, Feng Xu

**Affiliations:** 1 School of Management, Shandong University, Jinan 250199, China; chuying@mail.sdu.edu.cn (Y.C.); qiguijie@sdu.edu.cn (G.Q.); 2 School of Management Science and Engineering, Shandong University of Finance and Economics, Jinan 250199, China; xufeng@sdufe.edu.cn

**Keywords:** open innovation platform, performance-contingent material rewards, innovator behavior, incentive mechanism, cognitive evaluation theory

## Abstract

In recent years, enterprises have increasingly recognized the pivotal role of external users in driving product innovation. Open innovation platforms (OIPs), which facilitate interactions between companies and external innovators, have emerged as critical conduits in this regard. However, OIP managers face the challenge of motivating innovators to sustain their contributions. While some OIPs have implemented material incentives, the impact of such rewards on users’ ongoing innovation efforts remains uncertain. This study utilized a large-scale dataset from an OIP to examine how performance-contingent material rewards influence the subsequent behaviors of online innovators. Employing a quasi-experimental design involving propensity score matching (PSM) and difference-in-differences (DID) analysis, we found that receiving performance-contingent material rewards led to a decrease in the quantity of subsequent ideas generated by innovators. However, these rewarded innovators produced ideas of higher quality. Interestingly, the novelty of ideas submitted by innovators declined following their receiving of rewards. Moreover, newly enlisted innovators exhibited a more positive response to these incentives. Our findings provide valuable insights for platform managers seeking to optimize incentive mechanisms. We suggest adopting diversified incentive approaches and refining incentive strategies to effectively motivate continuous innovation among users on OIPs.

## 1. Introduction

OIPs are web-based community platforms established by companies to engage external users in product innovation and streamline innovation processes [1]. A significant challenge for platform managers is sustaining the continuous contribution of innovators, particularly those generating high-quality ideas [2]. In recent years, many OIPs have implemented material rewards to incentivize their innovators [3]. While numerous studies have explored the impact of material incentives on user-generated content (UGC) platforms [4,5], research specifically focusing on crowdsourcing platforms like OIPs is lacking. The extensive understanding of material incentives in information-based UGC platforms may not directly translate to crowdsourcing platforms due to fundamental differences between these platforms [6].

UGC platforms primarily facilitate opinion expression, information sharing, and knowledge exchange through material incentives aimed at encouraging users to produce valuable content [6]. In contrast, OIPs aim to stimulate and aggregate ideas that provide innovative solutions to companies, requiring users with professional expertise and strategic thinking [3,7]. Therefore, the psychological effects of rewards on OIP users significantly differ from those on UGC platform users.

Online platforms generally employ the following two types of incentive mechanisms: completion-contingent incentives, which uniformly reward individuals meeting specific contribution criteria, and performance-contingent rewards, which reward users based on the quality of their contributions [5,8]. As enterprise-led platforms designed to gather external innovation knowledge, OIPs prioritize high-quality ideas, recognizing that low-quality contributions are not only unproductive but also increase the platform’s managerial review workload. Hence, OIP incentives often utilize a centrally discretionary performance-contingent mechanism to foster user engagement and encourage valuable idea generation. Survey results indicate that performance-contingent material rewards offered by platforms effectively motivate users’ active participation [9]. However, there is a need for systematic research into the subsequent behaviors of users who receive such rewards.

The innovators who receive performance-contingent material rewards are pivotal contributors to the platform, and their sustained engagement is critical. This study investigates how these rewards influence recipients’ subsequent idea generation behavior, focusing on the following research questions: (1) Quantity of Ideas Contribution: Does the number of ideas contributed by innovators increase or decrease after receiving performance-contingent rewards? (2) Quality and Novelty of Ideas: How does the quality and novelty of ideas contributed by award recipients change following the receipt of rewards? (3) Cumulative Effects on New Users: Are the impacts of performance-contingent rewards particularly cumulative among new users?

Addressing these questions necessitates developing new theoretical insights. While receiving performance-contingent rewards intuitively suggests a positive impact on subsequent behaviors, their specific effects within OIPs remain uncertain. On one hand, recipients may increase their effort and participation due to recognition. Conversely, rewards might induce complacency, potentially reducing contributions. Similarly, the influence on idea quality and novelty can vary, potentially enhancing or diminishing these aspects depending on whether rewards prioritize goal achievement over creativity. Furthermore, we examine how reward impacts vary across members with different tenures, hypothesizing that new members may respond more keenly to centrally provided incentives. To empirically answer these questions, we analyze data from Xiaomi Community Subsequent sections review the pertinent literature, particularly leveraging Cognitive Evaluation Theory to inform our hypotheses. This study aims to provide comprehensive insights into the dynamics of reward systems in OIPs, offering implications for platform management and suggesting avenues for future research.

## 2. Theoretical Framework

### 2.1. Related Literature

Previous research on material incentive policies can be broadly categorized into two main areas. One line of inquiry examines the impact of introducing material incentive policies on organizational performance by analyzing performance trends before and after policy implementation. The other investigates the individual effects of receiving material incentives on subsequent behaviors post-policy. Specifically, these studies compare the behaviors of individuals before and after they receive rewards, with the incentive policy remaining constant throughout the study period. Our study aligns with this latter group of research.

Moreover, material incentive strategies are typically classified into completion-contingent and performance-contingent categories. Completion-contingent strategies reward individuals who meet task requirements based on explicit, predefined standards (e.g., achieving sales targets), while performance-contingent strategies emphasize exceptional performance, rewarding those who exceed established standards by delivering higher-quality outputs. In the context of OIPs, which enterprises primarily use to gather high-quality innovative ideas, performance-contingent rewards are commonly implemented. This study specifically examines the effects of performance-contingent material incentives in OIP settings.

Extensive research has documented how centrally provided material incentives impact individual behaviors or platform-level variables in online settings. Building upon the framework proposed by Yu et al. (2022) [5], we integrate these dimensions into a comprehensive 2 × 2 matrix (Table 1). This matrix synthesizes the existing literature on material incentives within online platforms, highlighting the specific contexts and outcomes examined by each study.

Prior studies have primarily examined the impact of material incentive policies in information-based UGC platforms [17,18], particularly online review platforms. For instance, in a randomized field experiment, Burtch et al. (2018) observed that reviewers, when eligible for completion-contingent material incentives, increased their review frequency without altering the length of their reviews [11]. Similarly, Wang et al. (2012) investigated the introduction of performance-contingent incentives in online review settings [15]. Yu et al. (2022), using a quasi-experimental design, studied behavior changes among reviewers following the receipt of performance-contingent rewards [5]. Their findings indicated an increase in the production of high-quality reviews post-reward without a subsequent change in review sentiment. In the realm of online Q&A platforms, Hsieh et al. (2010) found that users tended to answer more questions after receiving completion-contingent material rewards, although the quality of their answers did not significantly change [14].

In contrast, there is a paucity of literature on material incentives within crowdsourcing platforms like OIPs. Ikeda and Bernstein (2016), through a field experiment, examined the impact of completion-contingent material incentives on crowdsourcing platforms, noting that per-task payments diminished intrinsic motivation, slowed task completion, and potentially compromised overall task quality [12]. Building on the work of Bénabou and Tirole (2006), who argued that external rewards may undermine intrinsic motivation and lead to reduced effort [19], OIP platforms commonly utilize performance-contingent material incentives. For instance, platforms such as LEGO Ideas reward exceptional ideas with prizes and cash, while Xiaomi’s MIUI platform rewards users who contribute a certain number of high-quality ideas with prizes. Ho et al. (2015) conducted multiple behavioral experiments analyzing the introduction of performance-contingent rewards on crowdsourcing platforms, finding that such incentives motivated users to exert greater effort, resulting in higher-quality work [16].

Our research, situated in the lower-right quadrant of Table 1, systematically examines behavioral changes among OIP innovators following the receiving of rewards, thereby extending prior research findings. Notably, whereas existing studies on crowdsourcing platforms tend to be task-oriented, focusing on immediate and standardized tasks, OIPs are oriented towards innovation, addressing long-term challenges such as new product designs and technological advancements. Participants in OIPs typically possess professional expertise and are motivated by intrinsic factors such as enjoyment and altruism [20,21], contrasting with the transactional nature of task-oriented crowdsourcing platforms. Consequently, incentive systems in OIP environments aim to stimulate users’ intrinsic motivation. Prior research has explored various incentive measures in non-UGC platforms. For instance, Tafesse (2021) found distinct effects of emotions and information in crowdfunding platforms [22], while Liao et al. (2021) identified peer and manager feedback as significant factors that promote continuous engagement in OIPs [23]. Despite the recognized influence of non-material factors on user motivation in participation, there remains a gap in systematically investigating the impact of extrinsic material rewards within OIP platforms.

Although studies in offline environments indicate that performance-contingent rewards enhance idea quality and overall participation in innovation systems, they may also decrease the volume of ideas contributed per participant [24]. Our research employs a quasi-experimental design to empirically examine the effects of performance-contingent rewards on the quantity, quality, and novelty of ideas contributed by innovators on OIPs. Furthermore, we explore how the impact of these rewards varies among users with different tenures. This study contributes to the existing literature by shedding light on the influence of performance-contingent rewards on individual participation, particularly in environments where intrinsic motivation plays a significant role, such as gaming and leisure communities.

### 2.2. Cognitive Evaluation Theory 

Cognitive Evaluation Theory (CET) stands as a significant branch within motivational psychology, specifically addressing intrinsic motivation, which originates from an individual’s inherent interest in or enjoyment of an activity [25]. CET posits that human behavior is influenced by both intrinsic factors (stemming from internal sources such as curiosity, the need for challenge, and self-realization) and extrinsic factors (driven by external incentives like rewards, bonuses, or penalties) [26]. 

In this study, the innovators on OIPs receive performance-contingent material rewards from the platforms, which represent typical external motivating factors. However, innovative behavior is often driven by intrinsic motivation as well, such as personal interest and enjoyment in solving problems [27,28]. Therefore, CET provides a theoretical framework for investigating how external rewards influence intrinsic motivation and overall innovative behaviors.

Central to CET are two core psychological needs: autonomy and competence [29]. Autonomy refers to the sense of having choice, freedom, and control over one’s actions, while competence involves feeling capable of successfully completing tasks [30]. CET asserts that activities supporting these needs enhance intrinsic motivation, whereas those undermining them may diminish it. Furthermore, CET explores the potential impact of extrinsic rewards on intrinsic motivation, proposing a “crowding-out” effect [31]. This effect occurs when external rewards are perceived as controlling, thereby reducing an individual’s sense of autonomy and subsequently diminishing their intrinsic interest in the activity.

In practical applications, CET has influenced various fields, including education, organizational behavior, sports psychology, and game design [32,33,34]. By understanding how to effectively nurture intrinsic motivation, educators, managers, and designers can develop better teaching methods, incentive structures, and training programs that bolster individual engagement, effort, and overall performance [35]. This paper applies CET as a framework to investigate innovator participation in OIPs and develops hypotheses accordingly.

## 3. Hypothesis Development

This paper examines the impact of performance-contingent rewards on subsequent innovative behaviors among recipients. The study focuses on comparing the behaviors of innovators before and after receiving rewards, with the platform’s incentive policy remaining unchanged throughout.

### 3.1. Impact on Subsequent Idea Generation Quantity

Performance-contingent material rewards serve as clear signals to individuals that their contributions are valued by the organization, thereby enhancing the perceived value of their tasks [36]. According to CET, recognition of one’s efforts through external motivation can augment intrinsic motivation, thereby fostering increased participation [25]. Similarly, Yu et al. (2022) used a quasi-experimental design to demonstrate how performance-contingent material rewards on an online review platform can enhance participants’ engagement, resulting in increased idea contributions [5].

Conversely, the literature suggests that receiving performance-contingent rewards may lead to overconfidence and reduced persistence [37]. CET underscores the significance of autonomy in maintaining intrinsic motivation. If performance-contingent material rewards are perceived as exerting control over individuals’ creative processes, intrinsic motivation and ongoing participation may decline. Previous studies have highlighted hidden costs associated with rewards, such as shifting focus from task processes to reward attainment [38]. Moreover, rewards may diminish willingness to persist by directing attention towards performance outcomes rather than developmental progress.

Given these theoretical arguments and conflicting empirical evidence regarding the effects of performance-contingent rewards on idea quantity, we propose the following competing hypotheses:

**H1a:**  *Performance-contingent material rewards increase the number of ideas generated by award recipients.*

**H1b:**  *Performance-contingent material rewards decrease the number of ideas generated by award recipients.*

### 3.2. Impact on Subsequent Idea Quality

In contrast to information-based UGC platforms, OIPs require users to contribute high-quality ideas that not only reflect time and effort but also necessitate professional skills and knowledge [39]. Moreover, factors such as inspiration, community feedback, and guidance play pivotal roles in enhancing users’ innovation capabilities. As discussed in Section 3.1, performance-contingent material rewards can influence the intrinsic motivation of awardees through two contrasting pathways, depending on their perceived impact. Here, we consider two possible pathways to hypothesize how performance-contingent rewards affect the quality of subsequent contributions by awardees.

Firstly, according to CET, performance-contingent material rewards can serve as recognition of individual contributions and capabilities [40]. Receiving rewards can enhance perceived competence, typically leading to improved performance. For instance, Pink (2011) suggested that office workers may perform better after receiving such rewards [35]. In the context of OIPs, positive feedback from rewards could boost the confidence and self-efficacy of awardees, thereby enabling them to generate higher-quality ideas in future innovative endeavors. Moreover, receiving performance-contingent material rewards may confer an “expert” identity, facilitating increased social interactions and learning opportunities that could inspire new ideas.

Conversely, prior research indicates that external rewards may lead to detrimental behaviors such as reduced effort, thereby negatively impacting intrinsic motivation [19]. In OIPs, reliance on performance rewards might cause awardees to overlook the intrinsic value of participating in innovation. Given that generating high-quality ideas demands substantial effort and time, awardees under time constraints may prioritize quantity over quality when contributing ideas to meet performance criteria, potentially compromising idea quality. 

Hence, the impact of performance-contingent material rewards on the quality of ideas contributed by recipients remains uncertain. We therefore propose the following competing hypotheses:

**H2a:**  *Performance-contingent material rewards enhance the quality of ideas generated by award recipients.*

**H2b:**  *Performance-contingent material rewards reduce the quality of ideas generated by award recipients.*

### 3.3. Impact on Subsequent Idea Novelty

An intriguing question is how performance-contingent material rewards influence the novelty of ideas subsequently contributed by recipients. According to CET, if material rewards are perceived as being a recognition of individual capabilities and contributions, recipients may experience heightened feelings of competence [41]. Under these conditions, performance-contingent material rewards might encourage individuals to explore more novel ideas, feeling empowered to seek and realize these ideas freely and competently. Additionally, material rewards could offer practical support, enabling recipients to engage in more experimentation and exploration, thereby potentially increasing the novelty of their contributions.

Conversely, if material rewards are perceived as constraints on autonomy or as controlling mechanisms, intrinsic motivation could diminish [35]. When rewards are tied to specific behaviors, individuals may shift their focus from intrinsic interest and satisfaction to obtaining the reward itself [42]. Increased extrinsic motivation might lead to reduced idea novelty, as individuals may favor familiar, reward-proven paths over exploring unknown, innovative solutions. Moreover, some individuals might experience heightened pressure and expectations following rewards, which could impede the free flow of creative thinking and diminish the novelty of their ideas.

In summary, existing theories do not definitively predict how performance-contingent material incentives affect the novelty of subsequent ideas contributed by award recipients. Therefore, we pose the following hypotheses as open empirical questions:

**H3a:**  *Performance-contingent material rewards enhance the novelty of ideas generated by the award recipients.*

**H3b:**  *Performance-contingent material rewards reduce the novelty of ideas generated by the award recipients.*

### 3.4. Differential Impact on Innovators with Varying Tenures

Responses to material rewards vary significantly between long-tenured innovators and newcomers within a community, influenced by differences in motivation, experience, and commitment to, as well as identification with, the community [43,44,45]. 

Long-tenured users typically possess strong intrinsic motivation and a deep sense of community identification [46]. While they may appreciate material incentives as a form of recognition, these rewards may have a limited impact on their continued or increased participation. Their behavior tends to be driven more by intrinsic interests and a commitment to community values. Following the receiving of performance-contingent rewards, long-tenured users may seek to exert greater influence within the community through initiatives such as facilitating discussions, offering detailed guidance, or assuming leadership roles rather than focusing solely on subsequent rewards.

Conversely, for newcomers to the community, performance-contingent material rewards can significantly enhance their extrinsic motivation. This often results in increased engagement within the community, facilitating rapid accumulation of experience and elevation of their status. Moreover, receiving such rewards may motivate newcomers to establish a long-term commitment to the community, viewing their success as a validation of their efforts and a pathway to further achievements.

Based on the above analysis, we propose the following hypothesis:

**H4:**  *Performance-contingent material rewards have differing impacts on subsequent behaviors of long-tenured and short-tenured innovators, with long-tenured users exhibiting less pronounced effects.*

## 4. Research Design

### 4.1. Research Context

We conducted a quasi-experimental study on Xiaomi’s open innovation platform, which was established in 2011 as Xiaomi Community. Xiaomi is renowned for its innovative technologies, including high-end smartphones, internet TVs, and smart home ecosystems. The platform aims to collect external ideas to enhance innovation. To incentivize user contributions, Xiaomi Community operates a performance-contingent incentive program that rewards innovators who submit the highest-quality ideas each month.

Ideas on the platform are evaluated based on their innovation, feasibility, and alignment with company strategy. High-quality ideas adopted by managers contribute to the ranking of users, with top contributors receiving rewards such as Xiaomi electronics, peripherals, or vouchers ranging from CNY 10 to 50. Each month, up to 200 recipients are selected and announced with a thank you note that lists the winners. Notably, if multiple users submit identical ideas on the same day, only the first submission is counted towards performance metrics. Winners do not receive additional recognition (such as badges or titles) beyond the award announcement.

Data collection for our study spanned from November 2020 to October 2023 and encompassed detailed information on ideas (idea ID, innovator ID, submission date, adoption status, idea description, number of likes, peer comments, manager responses, device model, operating system) and innovators (innovator ID, tenure, etc.).

### 4.2. Variable Measurement

This study investigates the following three primary dependent variables: the quantity, quality, and novelty of ideas. Quantity is measured by the number of ideas generated by an innovator (i) in a month (t), denoted as ${IdeaCnt}_{it}$. Quality is assessed using two established metrics from prior research [47,48]. The first metric, ${AdoptRate}_{it}$, indicates the rate at which ideas submitted by an innovator (i) in a month (t) are adopted. The second metric, ${Likes}_{it}$, represents the average number of likes received by an innovator’s (i) ideas in a month (t).

To gauge the novelty of ideas, we employ ${IdeaNovelty}_{it}$, which measures the average novelty of ideas submitted by an innovator (i) in a month (t). Measuring novelty requires establishing a reference set. Following Burtch et al. (2022), we utilize the entire corpus of ideas generated during the observation period on the platform as the global vocabulary [6]. This approach ensures a comprehensive foundation for assessing novelty, as all ideas are publicly accessible and potentially replicable by other innovators.

The calculation of novelty employs the TF-IDF (Term Frequency-Inverse Document Frequency) method [49]:

1. **Calculate Term Frequency (TF):** Determine the frequency of each word within an idea, normalized by the idea’s length to mitigate biases stemming from varying idea lengths.
2. **Calculate Inverse Document Frequency (IDF):** Compute the logarithm of the inverse frequency of each word across all ideas, thereby assigning lower weights to common words and higher weights to rare words.
3. **Compute TF-IDF Values:** Multiply the TF of a word in a specific idea by its IDF, yielding the word’s TF-IDF value for that idea. Higher TF-IDF values indicate greater uniqueness and rarity within the entire corpus.
4. **Novelty Score:** Sum the TF-IDF values of all words in an idea to derive its novelty score. A higher total score signifies greater uniqueness and the use of less common terms, which is indicative of higher novelty.

The independent variables in our study are defined as follows: ${Treat}_{i}$ is a binary indicator that equals 1 if an innovator (i) belongs to the treated group; it equals 0 otherwise. ${After}_{t}$ is an indicator that equals 1 if the observation occurs after the treatment period; it equals 0 otherwise. The primary independent variable of interest is ${ReceivingReward}_{it}$, which equals 1 for observations where innovators receive the treatment after the treatment period; it equals 0 otherwise. Additionally, we include ${Tenure}_{it}$, representing the number of months since an innovator (i) joined the platform by month (t).

Given the potential influence of manager replies and peer feedback on user engagement, we control for these variables as well. ${ManagerReply}_{it}$ denotes the average number of replies received from managers by an innovator (i) in a month (t), while ${PeerFeedback}_{it}$ represents the average amount of feedback received from peers by an innovator (i) in a month (t).

### 4.3. Matching

To isolate the effect of receiving material rewards on subsequent innovator behavior, a direct approach is to compare behaviors before and after receiving the reward. However, this method has limitations due to potential natural changes in innovator behavior over time. To mitigate this issue, we employ PSM to create a control group that closely resembles the treated group in observable characteristics before the treatment period, thereby mimicking a randomized experimental design. Specifically, we identify a control innovator who, based on pre-treatment characteristics, is similar but did not receive the reward. All other conditions, such as interactions with platform managers, remain consistent for both treated and control innovators before and after treatment. By utilizing the control group, we account for any natural changes in behavior over time, allowing us to attribute observed differences to the impact of performance-contingent rewards. 

Individuals who received performance-contingent rewards between November 2021 and January 2022 are categorized as treated innovators. We excluded six innovators who received rewards multiple times to ensure clarity in identifying the treatment effect. Additionally, we focused our analysis on innovators with documented activity records preceding the award month, leveraging these records to match them with comparable control innovators. This selection process resulted in a sample of 553 rewarded innovators. Subsequently, we constructed a panel dataset at the innovator–month level, where each observation corresponds to an individual innovator for a specific month.

A key aspect of our identification strategy is the absence of a unified treatment start time within our dataset, as reward recipients received their awards in different months. During our observation period (November 2021 to January 2022), a total of three award presentations occurred. Consequently, we conducted three separate matches, each targeting awardees from one award cycle and non-awarded innovators from the same period. Our propensity score was calculated based on the following observable characteristics: (1) idea attributes reflecting the quality, quantity, and novelty of idea generation (i.e., ${IdeaCnt}_{i}$, ${AdoptRate}_{i}$, ${Likes}_{i}$, and ${IdeaNovelty}_{i}$); (2) interaction characteristics (ManagerReply it and PeerFeedback it); (3) innovator information, such as platform tenure and membership level before treatment (Tenure i and MemberLevel i). Specifically, these characteristics were assessed for each award recipient and their non-awarded counterparts six months prior to the award date.

Subsequently, we conducted one-to-one nearest neighbor matching without replacement, resulting in a sample of 1106 innovators who were evenly split into treatment and control groups (553 innovators each). To assess the effectiveness of our matching procedure, we performed a balance test on covariates between the treatment and control groups, presented in Table 2. Our findings, at a 95% confidence level (α = 0.05), indicate no significant differences in observable characteristics between the two groups before treatment. This ensures that the control group is effectively comparable to the treatment group prior to intervention.

Following PSM, we successfully created matched pairs between innovators who received the award and those who did not. Subsequently, we aligned these award-winning innovators with their respective control counterparts based on the month of award reception. Specifically, we selected a study period encompassing six months before and after the award date. Given the variation in award receival across months, we standardized each innovator’s participation period using a monthly index, ensuring the award month was consistently designated as the 0th month. This approach yielded a panel dataset spanning 12 months and comprising 553 pairs of innovators.

## 5. Empirical Analysis

### 5.1. Model-Free Evidence

Summary statistics for key variables of both groups of innovators before and after the award are presented in Table 3, with each treatment period covering six months. Comparatively, following receiving of the performance-contingent award, innovators exhibited a decrease in both the quantity and novelty of ideas while showing an increase in idea adoption rates and the number of likes received compared to control innovators.

**Figure 1:**
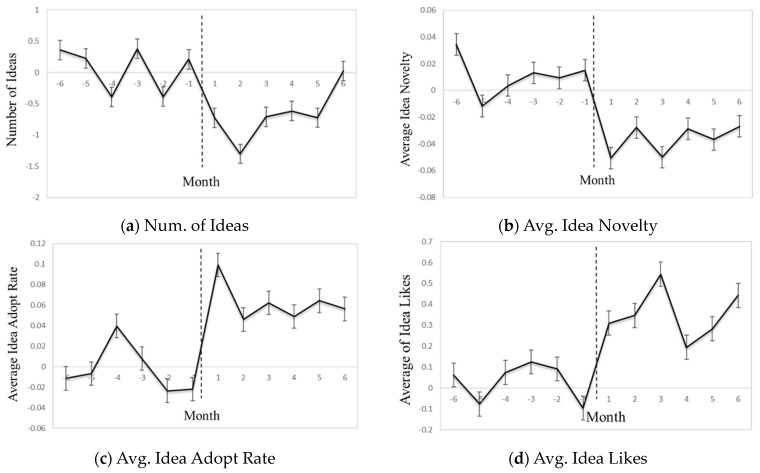
Innovator Behavior Across 12 Months (The dashed line is the month in which the user won the award).

Furthermore, we present model-free line plots depicting the mean differences in dependent variables between the treatment and control groups over a twelve-month period, illustrated in Figure 1. These plots offer non-model-based evidence regarding changes in innovators’ behaviors following the receipt of performance-contingent material rewards, broadly supporting our hypotheses. For example, the plots indicate a significant decrease in idea contributions among innovators after receiving rewards compared to those who did not receive rewards.

### 5.2. DID Estimation

#### 5.2.1. Econometric Framework

We employ panel DID analysis to test our hypotheses. DID is a widely-used econometric technique for evaluating quasi-experimental designs. This method assesses the treatment effect by comparing changes in outcomes between a treated group and a control group before and after the treatment period. In our study, we utilize DID to examine how the receipt of performance-contingent material rewards impacts innovative behavior.

(1) $${DV}_{it}=\beta_{0}+\beta_{1}\cdot {ReceivingReward}_{it}+\gamma \cdot {Controls}_{it}+\mu_{i}+\varphi_{t}+\varepsilon_{it}$$

Here, ${DV}_{it}$ denotes the dependent variables (e.g., ${IdeaCnt}_{it}$, ${AdoptRate}_{it}$, ${Likes}_{it}$, and ${IdeaNovelty}_{it}$) for an individual (i) at a certain time (t). The coefficient $\beta_{1}$ measures the average treatment effect of receiving rewards on subsequent innovative behavior. ${ReceivingReward}_{it}$ is an indicator variable that equals 1 if an innovator (i) receives rewards and 0 otherwise. $\mu_{i}$ captures innovator-fixed effects, while $\varphi_{t}$ captures time-fixed effects. ${Controls}_{it}$ denotes a vector of control variables. $\varepsilon_{it}$ is the error term accounting for unobserved factors affecting ${DV}_{it}$.

Our hypothesis explores the heterogeneous responses of innovators with varying tenures to performance-contingent rewards. To test this, we utilize the DID framework. Innovators are categorized into longer-tenured (${LongerTenure}_{i}$) and shorter-tenured (${ShorterTenure}_{i}$) groups based on whether their tenure exceeds or falls below the median tenure of all innovators in our sample. Building on previous studies [50], we introduce interaction terms between ${ReceivingReward}_{it}$ and these two tenure categories in Equation (2). Furthermore, we interact the innovator’s tenure category with control variables, as these factors may influence the innovation behaviors of longer- and shorter-tenured innovators differently. Time-fixed effects are also segmented into two groups corresponding to these tenure levels, acknowledging potential variations in their innovation behaviors over time. The resulting model is formulated as follows:

(2) $${DV}_{it}=\beta_{0}+\beta_{1}{\cdot ReceivingReward}_{it}\times {LongerTenure}_{i}+\beta_{2}{\cdot ReceivingReward}_{it}\times {ShorterTenure}_{i}+\gamma \;\cdot {Controls}_{it}+\mu_{i}+\varphi_{t}+\varepsilon_{it}$$

Our primary interest lies in estimating the coefficients *β*1 and *β*2 in Equation (2). *β*1 quantifies the effect of receiving performance-contingent rewards on the subsequent contributions of longer-tenured innovators, whereas *β*2 assesses this effect on shorter-tenured innovators. By comparing *β*1 and *β*2, we examine how the impact of receiving performance-contingent rewards varies across different tenures. It is important to note that the vector ${Controls}_{it}$ includes both control variables and interaction terms involving the two tenure categories.

#### 5.2.2. Hypothesis Testing

In the hypothesis testing section, our primary objective is to evaluate the influence of performance-contingent material rewards on subsequent innovator behaviors. Building upon the hypotheses outlined in Section 3.1, we conducted comprehensive analyses focusing on the quantity, quality, and novelty of ideas generated. Table 4 presents the impact of receiving performance-contingent rewards on overall innovator behaviors. Firstly, we find a significant negative impact of rewards on the number of ideas generated (*β* = −1.121, *p* < 0.05), thereby supporting H1b. This suggests that performance-contingent material rewards decrease the subsequent number of ideas contributed by recipients. Secondly, we examined changes in idea adoption rates and the number of likes as indicators of idea quality. Our results indicate a significant increase in both the average adoption rate (*β* = 0.064, *p* < 0.01) and the number of likes (*β* = 0.381, *p* < 0.01) following the receipt of performance-contingent rewards, supporting Hypothesis H2a. Thirdly, performance-contingent rewards exhibit a significant negative impact on the novelty of subsequently submitted ideas (*β* = −0.044, *p* < 0.01), corroborating Hypothesis H3b. This suggests that awardees may prioritize idea adoption over innovation novelty after receiving rewards. These findings imply that, while performance-contingent rewards enhance certain aspects of innovator behavior, such as idea adoption and popularity, they may inadvertently suppress the generation of new and innovative ideas.

Table 5 delves deeper into the effects of performance-contingent rewards on innovators based on their tenure. We categorized innovators into the following two groups: those with longer tenures, where tenure on the platform exceeded the median, and those with shorter tenures. Our findings reveal that the impact of performance-linked material rewards on innovators with longer tenures is comparatively weaker than on those with shorter tenures. Specifically, regarding the number of ideas and the novelty of ideas, innovators with longer tenures did not show significant changes in these metrics after receiving rewards. This suggests that newer users are more responsive to material incentives, thereby corroborating Hypothesis H3. These results underscore that while performance-contingent rewards can spur engagement among newer users, their effect diminishes among more established innovators, particularly in terms of idea generation and novelty.

#### 5.2.3. Relative Time Model

We employ a Relative Time Model (RTM) to examine whether our data satisfy the parallel trends assumption. An advantage of a RTM is its ability to not only validate the pre-treatment parallel trends assumption but also to capture the dynamics of treatment effects [6]. Given that the impact of rewards may materialize immediately after recipients receive them, we establish relative time −1 as the baseline to assess parallel trends. Table 6 presents the difference estimates of outcome variables between the treatment and control groups at various relative time points. Prior to treatment, we find no statistically significant differences between the two groups, indicating that the parallel trends assumption holds. However, following the treatment, we observe a series of significant coefficient changes, suggesting that performance-contingent rewards influence innovator behavior. Importantly, the estimates derived from the RTM align consistently with those obtained using the DID method.

### 5.3. Robustness Checks

To ensure the robustness of our findings, we conducted several additional analyses. Table 7 provides a summary of these robustness tests. Importantly, the results from these tests align closely with our main findings.

## 6. Discussion

This study explores the influence of performance-contingent material rewards on subsequent innovative behaviors among online innovators within an OIP. Using empirical data from Xiaomi Community, we analyze how these rewards impact the quantity, quality, and novelty of the generated ideas. Our findings demonstrate that performance-contingent material rewards have a significant effect on innovators’ idea generation. Specifically, these rewards lead to a reduction in the quantity of ideas produced by recipients. However, they simultaneously enhance the adoption rate and receive more likes, indicating an improvement in idea quality. Interestingly, the mechanism tends to diminish the novelty of ideas, possibly because innovators prioritize meeting reward criteria over exploring more unconventional ideas. Furthermore, our analysis uncovers variations in reward impact based on innovator tenure. We observe that the effect of performance-contingent material rewards is less pronounced among long-tenured innovators compared to their short-tenured counterparts. This suggests that newer users are more motivated by such rewards while longer-tenured innovators may be driven more by intrinsic motivations. In conclusion, this study provides insights into how performance-contingent material rewards influence innovation within OIPs, highlighting nuanced effects on idea quantity, quality, and novelty, as well as the differential responses based on innovator tenure.

### 6.1. Theoretical Contributions

This study contributes significantly to the fields of open innovation, motivation theory, and online platform management. It provides novel insights into the effects of performance-contingent material rewards on online innovators.

Firstly, previous research in open innovation contexts has predominantly highlighted the positive impacts of monetary incentives on participation rates and output quantity. However, this study advances our current understanding by demonstrating a nuanced relationship, indicating that, while performance-contingent material rewards may decrease the sheer quantity of ideas generated by innovators, they concurrently enhance the quality of those ideas. This finding challenges the conventional assumption of an inherent correlation between quantity and quality, underscoring the necessity of considering both dimensions when designing incentive schemes.

Secondly, the study explores the intricate effects of incentives on idea novelty. It reveals a decline in idea novelty following the receipt of rewards, shedding light on a less-examined facet of incentive mechanisms in open innovation platforms. This suggests that, while material incentives can incentivize efforts to refine existing concepts, they might unintentionally discourage risk-taking and experimentation—essential elements for fostering genuinely novel ideas. This discovery enriches the theoretical framework by emphasizing the importance of balancing short-term productivity gains with long-term innovation potential.

Furthermore, the study identifies differentiated responses across innovator cohorts. It also finds that newly recruited innovators respond more positively to performance-contingent rewards compared to established innovators. This insight contributes to the growing literature on the role of social and psychological factors in shaping innovation behavior, highlighting that personal and situational characteristics significantly influence the effectiveness of incentive strategies.

### 6.2. Practical Implications

This study investigates the influence of performance-contingent material rewards on the subsequent innovative behaviors of online innovators within an OIP, offering two practical insights. Firstly, it informs the design of incentive mechanisms. The findings demonstrate that performance-contingent material rewards positively enhance idea quality while potentially exerting a negative impact on idea novelty. This provides crucial guidance for OIP managers in devising and adjusting incentive structures. It suggests that managers should consider the multidimensional effects of incentives to ensure that they foster innovators’ enthusiasm without stifling their creative thinking. Diverse incentive schemes, such as integrating material rewards with social recognition or offering further developmental opportunities, could comprehensively promote innovators’ contributions.

Secondly, the study underscores the importance of tailored incentive strategies. Previous studies have indicated that entrepreneurs or managers should adopt differentiated means based on different target groups [22]. This study reveals varying impacts of performance-contingent material rewards on innovators with different tenures, implying the need for customized incentive approaches based on innovators’ backgrounds and participation motivations. For newly onboarded innovators, material rewards serve as effective incentives to swiftly engage them in participation and contributions. In contrast, long-term participants may benefit more from strategies that nurture intrinsic motivation and foster community spirit. This makes it necessary for OIP managers to develop nuanced and personalized incentive strategies that cater to the distinct characteristics and motivations of innovators, thereby effectively motivating diverse groups of participants.

## 7. Conclusions

In conclusion, this study provides a nuanced understanding of the effects of performance-contingent material rewards on the subsequent innovation behaviors of online innovators within an OIP context. To rigorously investigate the underlying mechanisms in complex phenomena, we formulated multiple sets of competing hypotheses rooted in cognitive assessment theory and informed by the existing literature. Our analysis, leveraging a large-scale dataset and employing quasi-experimental methods, reveals that, while such rewards may reduce the quantity of ideas generated by rewarded innovators, they concurrently enhance the quality of these ideas. Notably, the novelty of submitted ideas declines following reward receipt, suggesting a potential trade-off between creativity and reward-driven refinement. Furthermore, newly recruited innovators respond more favorably to these incentives, indicating that the effectiveness of material rewards may vary across different user segments. These findings have important implications for OIP managers seeking to optimize their incentive mechanisms. We recommend adopting a diversified approach to incentives, refining strategies to strike a balance between quantity and quality of contributions, and tailoring incentives to cater to the diverse needs and motivations of innovators. Ultimately, our research contributes to the broader discourse on motivating continuous innovation in open collaboration settings.

Our research has several limitations that also present opportunities for future studies. Firstly, this study did not extensively investigate the mechanisms underlying these behavioral changes. Future research could employ qualitative methods like in-depth interviews or surveys to further explore the interaction between agency and structure in terms of sense-making and sense-giving [51]. Such an approach could offer deeper insights into how innovators make and give sense to their innovative activities within specific social contexts, shedding light on their psychological states and motivations. Secondly, this study focuses primarily on a specific OIP, namely Xiaomi Community, which may constrain the generalizability of the findings. Given potential variations in how different types of online platforms respond to performance-contingent material rewards, conducting cross-platform comparative studies would be beneficial. Such studies would not only validate the applicability of the findings across platforms but also elucidate how distinct platform characteristics influence the efficacy of reward mechanisms. Lastly, future research could investigate the impact of different types (e.g., cash, vouchers, electronic products) and levels of material incentives on innovators’ participation and output. This exploration could assist platform managers in optimizing incentive policies by identifying the most cost-effective and motivating incentive approaches.

## Figures and Tables

**Table 1:** Summary of Material Incentives Research on Online Platforms.

		Focal Point of the Study	
		The Impact of Introducing the Material Incentive Policy	The Impact of Receiving Material Rewards
Incentive Mechanism	Completion-Contingent	Online Review Platforms: (Khern-am-nuai et al. 2018) [10]; (Burtch et al. 2018) [11] Online Q&A Platforms: (Kuang et al. 2019) [4] Online Crowdsourcing Platforms: (Ikeda and Bernstein 2016) [12]	Online Review Platforms: (Qiao et al. 2017) [13] Online Q&A Platforms: (Hsieh et al. 2010) [14]
	Performance-Contingent	Online Review Platforms: (Wang et al. 2012) [15] Online Crowdsourcing Platforms: (Ho et al. 2015) [16]	Online Review Platforms: (Yu et al. 2022) [5]

**Table 2:** PSM Balancing Test for Covariates.

Variables	Mean (Treatment)	Mean (Control)	*p*-Value
$IdeaCnt_{i}$	20.60	17.22	0.107
$AdoptRate_{i}$	0.37	0.36	0.513
$Likes_{i}$	1.86	1.89	0.655
$IdeaNovelty_{i}$	0.48	0.49	0.644
$ManagerReply_{i}$	0.41	0.42	0.681
$PeerFeedback_{i}$	0.54	0.52	0.417
$MemberLevel_{i}$	3.05	2.96	0.345
$Tenure_{i}$	1466.90	1389.45	0.186

**Table 3:** Summary Statistics.

Variables		Panel A: Pre-Treatment Period (6 Months)					Panel B: Post-Treatment Period (6 Months)				
		Mean	Std. Dev.	Min	Max	Obs	Mean	Std. Dev.	Min	Max	Obs
$IdeaCnt_{it}$	1	20.60	36.869	1	547	448	11.898	21.363	1	315	400
	2	17.22	25.346	1	187	461	13.58	22.282	1	223	461
$AdoptRate_{it}$	1	0.37	0.265	0	1	448	0.42	0.292	0	1	400
	2	0.36	0.310	0	1	461	0.36	0.273	0	1	461
$Likes_{it}$	1	1.86	1.050	0	7	448	2.15	1.420	0	13	400
	2	1.89	1.287	0	6	461	1.78	1.009	0	5	461
$IdeaNovelty_{it}$	1	0.48	0.169	0.04	0.89	448	0.44	0.182	0.02	0.87	400
	2	0.49	0.199	0.01	0.89	461	0.48	0.185	0.001	0.87	461
$ManagerReply_{it}$	1	0.41	0.261	0	1	448	0.37	0.264	0	1	400
	2	0.42	0.324	0	1	461	0.36	0.254	0	1	461
$PeerFeedback_{it}$	1	0.54	0.270	0	1.13	448	0.47	0.283	0	2	400
	2	0.52	0.320	0	1.12	461	0.48	0.256	0	1	461
$Tenure_{it}$	1	48.54	29.409	2	89	448	54.84	29.409	8	95	400
	2	45.40	29.808	1	89	461	51.40	35.808	7	95	461

Note: Group 1 is the treatment group. Group 2 is the control group.

**Table 4:** Impact of Receiving Performance-Contingent Rewards.

Variables	${IdeaCnt}_{it}$	${AdoptRate}_{it}$	${Likes}_{it}$	${IdeaNovelty}_{it}$
${ReceivingReward}_{it}$	−1.121 ** (0.454)	0.064 *** (0.017)	0.381 *** (0.090)	−0.044 *** (0.016)
${ManagerReply}_{it}$	0.583 *** (0.189)	0.558 *** (0.012)	0.288 *** (0.078)	0.024 ** (0.011)
${PeerFeedback}_{it}$	0.135 *** (0.201)	0.354 *** (0.013)	0.205 *** (0.071)	0.020 ** (0.010)
Fixed Effects	Yes	Yes	Yes	Yes
Obs	5378	5378	5378	5378
Adj. R^2^	0.086	0.467	0.027	0.009

Standard errors in parentheses are robust. *** *p* < 0.001, ** *p* < 0.01.

**Table 5:** Differential Impact by Tenure.

Variables	${IdeaCnt}_{it}$	${AdoptRate}_{it}$	${Likes}_{it}$	${IdeaNovelty}_{it}$
${ReceivingReward}_{it}\times {LongerTenure}_{i}$	−0.434 (0.569)	0.060 *** (0.023)	0.352 *** (0.129)	−0.028 (0.023)
${ReceivingReward}_{it}\times {ShorterTenure}_{i}$	−1.992 ***	0.074 *** (0.024)	0.439 *** (0.128)	−0.061 *** (0.022)
${Controls}_{it}\times {LongerTenure}_{i}$	Yes	Yes	Yes	Yes
${Controls}_{it}\times {ShorterTenure}_{i}$	Yes	Yes	Yes	Yes
Fixed Effects	Yes	Yes	Yes	Yes
Obs	5378	5378	5378	5378
Adj. R^2^	0.096	0.396	0.030	0.012

Standard errors in parentheses are robust. *** *p* < 0.001.

**Table 6:** Estimation results of the Relative Time Model.

Variables	${IdeaCnt}_{it}$	${AdoptRate}_{it}$	${Likes}_{it}$	${IdeaNovelty}_{it}$
Relative time −6	−0.582 (1.154)	0.014 (0.040)	0.421 (0.200)	−0.060 (0.037)
Relative time −5	−0.489 (0.852)	−0.003 (0.033)	0.214 (0.172)	−0.025 (0.032)
Relative time −4	−0.695 (0.792)	0.017 (0.031)	0.175 (0.157)	−0.026 (0.030)
Relative time −3	−0.039 (0.723)	0.022 (0.028)	0.205 (0.153)	−0.010 (0.028)
Relative time −2	−0.242 (0.546)	−0.035 (0.026)	0.110 (0.139)	−0.013 (0.025)
Relative time −1	The baseline			
Relative time +1	−1.616 *** (0.561)	0.094 *** (0.029)	0.550 *** (0.154)	−0.070 ** (0.027)
Relative time +2	−1.534 *** (0.565)	0.095 *** (0.030)	0.528 *** (0.169)	−0.076 ** (0.030)
Relative time +3	−1.599 *** (0.596)	0.067 ** (0.031)	0.644 *** (0.167)	−0.057 * (0.030)
Relative time +4	−1.361 ** (0.561)	0.034 (0.034)	0.321 * (0.199)	0.041 (0.031)
Relative time +5	−1.054 (0.717)	0.043 (0.034)	0.575 *** (0.202)	−0.046 (0.034)
Relative time +6	−0.513 (0.886)	0.016 (0.035)	0.504 ** (0.241)	−0.050 (0.037)
Controls	Yes	Yes	Yes	Yes
Fixed Effects	Yes	Yes	Yes	Yes
Obs	5378	5378	5378	5378
Adj. R^2^	0.087	0.474	0.028	0.011

Note: Standard errors in parentheses. * *p* < 0.10, ** *p* < 0.05, *** *p* < 0.01. The Relative Time Model validates the DID approach by showing no significant differences before the treatment and demonstrating the treatment impact after receiving performance-contingent material rewards.

**Table 7:** Robustness Checks Summary.

	Description
Test1	We considered an alternative control group selection mechanism by using less strict matching criteria (i.e., fewer matching covariates) to obtain more matching pairs.
Test2	We used coarsened exact matching (CEM), an alternative matching algorithm, instead of PSM in the matching process.
Test3	We included innovators who received rewards multiple times during the study period.
Test4	We conducted a cross-validation-treating user tenure as a continuous moderating variable.
Test5	We performed placebo tests using bootstrap simulations to randomly reassign treatment.

## Data Availability

Data are contained within the article.
